# Using a Bayesian Network to Predict L5/S1 Spinal Compression Force from Posture, Hand Load, Anthropometry, and Disc Injury Status

**DOI:** 10.1155/2017/2014961

**Published:** 2017-10-01

**Authors:** Richard E. Hughes

**Affiliations:** Departments of Orthopaedic Surgery, Biomedical Engineering, and Industrial & Operations Engineering, University of Michigan, Ann Arbor, MI 48109, USA

## Abstract

Stochastic biomechanical modeling has become a useful tool most commonly implemented using Monte Carlo simulation, advanced mean value theorem, or Markov chain modeling. Bayesian networks are a novel method for probabilistic modeling in artificial intelligence, risk modeling, and machine learning. The purpose of this study was to evaluate the suitability of Bayesian networks for biomechanical modeling using a static biomechanical model of spinal forces during lifting. A 20-node Bayesian network model was used to implement a well-established static two-dimensional biomechanical model for predicting L5/S1 compression and shear forces. The model was also implemented as a Monte Carlo simulation in MATLAB. Mean L5/S1 spinal compression force estimates differed by 0.8%, and shear force estimates were the same. The model was extended to incorporate evidence about disc injury, which can modify the prior probability estimates to provide posterior probability estimates of spinal compression force. An example showed that changing disc injury status from false to true increased the estimate of mean L5/S1 compression force by 14.7%. This work shows that Bayesian networks can be used to implement a whole-body biomechanical model used in occupational biomechanics and incorporate disc injury.

## 1. Introduction

Stochastic modeling has become a useful analytical method in biomechanics over the last twenty years. Applications have included lumbar region muscle forces [1], musculoskeletal injury risk prediction [2–4], upper extremity joint mechanics [5–8] muscle modeling [9], population morphological modeling [10], probabilistic sensitivity analyses [11], tissue repair [12], and orthopaedic implant design [13, 14]. Past stochastic biomechanical models use Monte Carlo simulation, advanced mean value theorem, or Markov chain models. Unfortunately, these methods are all “forward” simulation methods that produce probability estimates of outcomes based on model inputs represented as random variables.

Bayesian networks are well established in artificial intelligence [15, 16], risk analysis [17], and reliability engineering [18–21] and have the potential to enhance biomechanical modeling. A Bayesian network is a graphical probabilistic model containing nodes and directed edges that can be used to compute probabilities when evidence has been entered at a variety of nodes in the graph, some of which are descendants of the primary variable of interest [22]. Consider the problem of predicting lumbar spine compression force during lifting, which is a common task in occupational biomechanics. Existing biomechanical models require task-related inputs like mass in the hands, anthropometry, and joint angles to predict compression force [23, 24]. Physiologically driven models also require electromyographic measurements [25–28]. The results of these models are predictions of spinal compression and shear forces. In theory, a Bayesian network could be used to also incorporate intervertebral disc injury status, which existing models cannot.

Therefore, the purpose of this project was to evaluate the feasibility of implementing a well-established static two-dimensional biomechanical model of lifting as a Bayesian network and extend it to include disc prolapse as a model input.

## 2. Methods

The design of this study consisted of five steps: (1) implement a well-established deterministic static two-dimensional biomechanical model for predicting L5/S1 shear and compression force as a Bayesian network, (2) extend the Bayesian network to include uncertainty in model inputs, (3) verify the implementation, (4) augment model to include disc injury status, and (5) evaluate effect of including disc injury status on L5/S1 compression force estimates.

A well-established deterministic model for predicting L5/S1 compression force during lifting was selected as the basis for this model [29] (pp. 130-131). This model computes intersegmental reaction forces and moments at the elbow, shoulder, and L5/S1 disc. Body segment lengths and masses are scaled from status and total body mass, respectively. The location of the center of mass for each segment is scaled to segment length. All necessary body segment anthropometric parameters are described in Chaffin et al. [29] (pp. 41–47). The model allows for a downward directed hand force representing the holding of an object in the hands. The model is bilaterally symmetric. A 50th-percentile female (161.8 cm stature and 65.6 kg body mass) was used for all simulations. The erector spinae moment arm was 5.3 cm according to Chaffin et al.'s recommendation for models not incorporating intra-abdominal pressure (p. 134).

The deterministic and stochastic versions of the lifting model were also coded in MATLAB R2014a (The Mathworks, Natick, MA) for purposes of validation. Monte Carlo simulation was used for the stochastic simulation, and each simulation contained 10^7^ iterations. Hand calculations were also performed for selected deterministic cases for code verification.

The next step was to implement the same model as a Bayesian network [30] using AgenaRisk software (Agena, Cambridge, UK). A Bayesian network is a graphical probabilistic modeling framework developed initially in artificial intelligence [15, 16]. A Bayesian network is a directed acyclic graph in which the directed edges represent conditional independence assumptions and node probability tables are associated with each node. Each node represents a random variable. The probability table associated with a node consists of a full statement of conditional probabilities for all nodes that are parents of the node. It is also possible to code deterministic relationships between nodes. Probability distributions can be defined for nodes, as well as observed values of the random variables. Junction tree methods are used to propagate probability distributions through the network [31].

**Figure 1:**
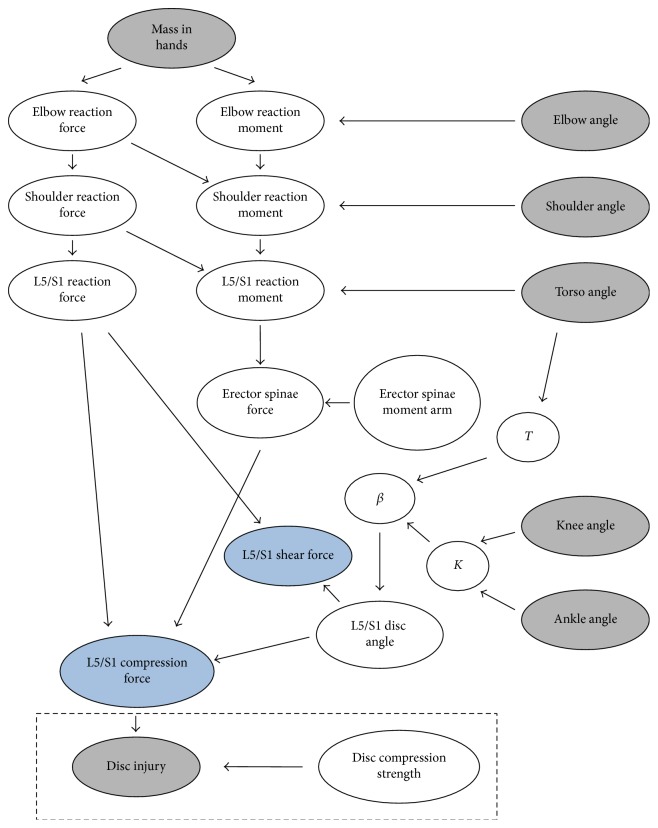
Bayesian network implementation of a two-dimensional top-down lifting model described by Chaffin et al. [29] (pp. 130–134). The “basic model” is the DAG outside the dashed rectangle at the bottom; the “injury-augmented model” is the entire DAG including the two nodes and edges inside the dashed rectangle at the bottom. Input variables are represented as gray nodes (*mass in hands*, *elbow angle*, *shoulder angle*, *torso angle*, *knee angle*, and *ankle angle*). The angles are defined relative to the horizontal. Intermediate angles *T* (torso angle from vertical) and *K* (included knee angle) are computed from input angles to reduce the complexity of the expression in *β*, which was the deviation of the disc angle from 40°. This was done to facilitate computation. Joint reaction forces and moments are calculated for the elbow, shoulder, and L5/S1 levels because it is a top-down modeling approach. The line of action of the erector spinae muscle is assumed to be perpendicular to the L5/S1 disc, so there is no directed edge from *erector spinae force* to *L5/S1 shear force*. The *erector spinae moment arm* is treated as a constant (5.3 cm) and has its own node. The variables to be predicted in this model are the *L5/S1 compression force* and *L5/S1 shear force*, and they are denoted as blue nodes. The injury-augmented model was created by adding the nodes contained in the dashed rectangle (*disc injury* and *disc compression strength*); these implement the inference model of disc failure based on structural reliability modeling. Because the *disc injury* node was an input in the injury-augmented model, its node is shaded gray.

In the Bayesian network implementation of the lifting model, nodes for input data were defined: *mass in hands*, *elbow angle*, *shoulder angle*, *torso angle*, *knee angle*, and *ankle angle* (Figure 1). Note that in this paper, variables are indicated in italics. Intermediate nodes were also defined for purposes of computation. Nodes were introduced for the reaction forces and moments at the elbow (*elbow reaction force and elbow reaction moment*), shoulder (*shoulder reaction force* and *shoulder reaction moment*), and L5/S1 level (*L5/S1 reaction force* and *L5/S1 reaction moment*). A node was created for the *erector spinae moment arm*, and another for *erector spine force*. The effect if intra-abdominal pressure was excluded because of the nonlinearity of the relationship between hip moment and pressure (p. 132). A node for *L5/S1 disc angle* was included, as were three nodes (*β*, *T*, and *K*) to implement a regression model for predicting deviation of disc angle from 40 degrees [32], which is how Chaffin et al. compute disc angle (p. 132). Two output nodes were defined: (1) *L5/S1 compression force* and (2) *L5/S1 shear force*. Edges were introduced to link the nodes in a way that represented mechanics. In AgenaRisk, each arc was given the deterministic relationships linking nodes. This defined the deterministic version of the lifting model.

The model was extended to a stochastic model by modifying the input nodes to be normally distributed random variables rather than deterministic values. Liu et al. [33] showed that assessment of body segment angles for a lifting model using common ergonomics tools has measurement error and these error estimates were used to define the standard deviation for each joint angle. Since Liu et al. [33] reported mean absolute errors, normality was assumed and the result of Geary [34] was used to compute standard deviation for the elbow, shoulder, and torso angles. The standard deviation for the error across all joints was also computed, and this was used for the knee and ankle.

The method was evaluated on a lifting task obtained from a study of heavy work that has been previously reported [35]. The task involved lifting chunks of carbon that had broken off large anodes in an aluminum smelter. Posture was collected from video of the job taken from a single viewpoint. The postural measurements were subject to error as shown by Liu et al. [33], and hand load was random because the carbon pieces broke off from anodes by a process that created a wide distribution of masses. Therefore, both posture and hand load were modeled as normally distributed random variables.  Table 1 provides the mean (and standard deviation) joint angles. The mass of the object in the hands was modeled as a normally distributed random variable having mean and standard deviation of 53.1 kg and 12.8 kg, respectively.

A modified model was developed that incorporates intervertebral disc prolapse status (a so-called “injury-augmented” model). It is the full DAG shown in Figure 1. It was developed by adding a probabilistic inference model of structural failure in which failure occurs when the applied load exceeds the compressive strength. Both applied load and strength were modeled as random variables [36]. Therefore, a node for intervertebral disc compressive strength was added. The distribution of compressive strength was assumed to be normally distributed. The mean (5448 N) and standard deviation (2366 N) values were taken from reports of cadaver testing [37]. A disc prolapse Boolean variable node was added that took on a true value if and only if the compression force exceeded the disc compressive strength. For the numerical example analyzed to illustrate the method, separate simulations were performed for three injury conditions: (1) no *disc injury* evidence entered, (2) *disc injury* Boolean variable set to false, and (3) *disc injury* Boolean variable set to true. The model also included modeling postures and hand loads as random variables as described above.

## 3. Results

The deterministic model implemented as a Bayesian network produced L5/S1 compression force estimates that were within 1.4 N (0.04%) of the MATLAB implementation predictions (Table 2). Similarly, both deterministic model versions produced the same L5/S1 shear force estimate. This demonstrates verification of the deterministic implementation. The Bayesian network basic model in which the joint angles were modeled as random variables produced greater average L5/S1 compression force than the MATLAB implementation. However, the difference in means was only 28.5 N (0.8%). When hand load was simulated as a random variable, the difference in means was only 3.8 N (0.1%). For the random posture case, the 25th percentile, median, and 75th percentile of the compression force distribution were within 290.2 N (4.5%), 1.6 N (0.05%), and 261.9 N (7.0%) of the MATLAB code Monte Carlo implementation results.

The injury-augmented model's predicted *L5/S1 compression force* changed as evidence was entered at the injury node. When evidence of no injury (*disc injury* = false) was entered at the injury node, the mean compression force was 3190.2 N (SD 789.3). However, when evidence of injury (*disc injury* = true) was entered, the predicted mean compression force increased to 3660.9 N (SD 820.0). This was an increase of 470.7 N (14.7%). Moreover, the injury evidence shifted the entire distribution. The 25th percentile value went from 2664.1 N to 3095.9 N when *disc injury* was changed from false to true; the 75th percentile similarly increased from 3690.1 N to 4180.1 N going from false to true. Predicted mean *L5/S1 shear force* also changed from 673.1 N (SD 108.9) to 715.7 N (SD 111.0) when *disc injury* was changed from false to true.

## 4. Discussion

This project demonstrated the ability of implementing a biomechanical force prediction as a Bayesian network. The error between mean L5/S1 compression force estimates was small (<1%), and it was lower when modeling hand load as a random variable than when modeling joint angles as random variables. While Bayesian networks have had limited use in biomechanical data analysis, they have not been used to implement whole-body biomechanical models.

A Bayesian network is a directed acyclic graph (DAG) whose nodes encode conditional probability statements and edges reflect conditional independence assumptions. Directed edges can be thought of as indicating causal relationships [38]. Directed paths in the DAG can also represent causal structures. In the first model developed (Figure 1), a DAG is constructed so that directed paths go from input variables (*mass in hands*, *elbow angle*, etc.) and internal structural variables (*erector spinae moment arm*, *L5/S1 disc angle*, etc.) to low back kinetic variables (*L5/S1 shear force* and *L5/S1 compression force*). This “basic model,” which merely implements the standard model of Chaffin, is the DAG shown outside of the dashed rectangle in Figure 1. Calculations on this network are straightforward and do not require Bayes' theorem. However, the full power of a Bayesian network is apparent when Bayes' theorem is used to calculate probabilities backwards along directed paths. The injury-augmented model (entire network in Figure 1) does this. Moreover, the injury-augmented model uses both the input data required for the basic model (Figure 1) and disc prolapse status (*disc injury* node in Figure 1 denoting the Boolean variable describing whether the person being analyzed has a prolapsed disc or not). The difference was 14.7%, which demonstrates the impact incorporating disc injury status can have on spinal compression force estimates.

Another interesting feature of calculations performed on a Bayesian network is how the *disc injury* variable affects *L5/S1 shear force*. Note that the *disc injury* node is a descendant of the L5/S1 shear force node, yet changing *disc injury* from false to true increases the mean L5/S1 shear force from 673.1 N to 715.7 N. If it is a descendant, how can it affect L5/S1 shear force? It is because there is—in terminology from graphical causal modeling—a “trek” from *disc injury* to L5/S1 shear force that does not contain a collider node [39]. Changing the value *of disc injury* from false to true affects *L5/S1 reaction force* through *L5/S1 compression force* because of Bayes' theorem. In turn, *L5/S1 reaction force* affects *L5/S1 shear force*. A similar effect could occur via the *L5/S1 disc angle* node.

Existing stochastic simulation methods are forward methods that propagate probability from distributions of input parameters to distributions of outputs. Previously, Monte Carlo simulation has been the most common tool for introducing probabilistic elements into deterministic biomechanical models [1–4, 7, 8, 13, 14, 40]. While intuitively easy to implement, Monte Carlo techniques are computationally intensive. Laz et al. [14] introduced the use of the advanced mean value theorem approach to probabilistic simulation in biomechanics. Markov chain models have also been used for stochastic modeling of soft-tissue failure [12]. In Bayesian network parlance, all of these methods place the input variables on nodes that are ancestors of the variable to be predicted. Variables that are graph-theoretic descendants of the predicted variable cannot influence the predictions. This makes it impossible to include injury status into the calculation of spinal compression and shear forces using existing probabilistic methods.

The most significant limitation of the Bayesian network implementation of the model was the difficulty in predicting the tails of the spinal compression force distribution. Specifically, there was a difference of 7% for the 75th percentile. An additional limitation was that the Bayesian network produced a better estimate of the mean compression force when the hand load was modeled as a random variable than when the joint angles were random. This is because the effect of joint angles on spinal compression force is nonlinear.

Comparisons of these results to other Bayesian network models in biomechanics are difficult because so few applications of Bayesian networks exist in biomechanics. Van Gestel et al. [41] and Lo et al. [42] used a Bayesian network for gait classification, but neither used the Bayesian network to implement a biomechanical model. Ma et al. [43] used a Bayesian network to analyze spinal kinematics in patients having low bone mineral density. Again, no biomechanical model was used. Bayesian networks have also been used in the analysis of clinical data related to degenerative disease of the lumbar spine. Takenaka and Aono [44] used a Bayesian network to develop a classifier for lower extremity muscle strength for patients presenting with drop foot following lumbar spinal decompression surgery.

A different comparison to existing literature can be made by noting the similarity to work done in the structural reliability area. The model developed here considers disc prolapse to occur when the applied load exceeds the compressive strength of the intervertebral disc to withstand load [36]. In this context, the current model can be viewed as using a stochastic biomechanical model to predict the load and cadaver testing to provide strength data. Thus, there is a conceptual connection to work implementing interference models in structural reliability models using Bayesian networks [45]. Donnell et al. [12] have also used the interference model of structural failure in biomechanics, although not implemented as a Bayesian network.

This model takes a simplistic view of spinal injury mechanics to highlight the Bayesian network modeling technique; future work should include subtle factors. The disc compression limit came from a study [37] that tested spinal motion segments in mild flexion, matching the lifting posture simulated. However, it used a single load to failure test paradigm, where other authors argue that cyclic loading is a very important factor in intervertebral disc failure [46, 47]. The current model also only considered compression in disc injury; the effects of shear should also be investigated [48].

In conclusion, this is a novel contribution to low back modeling because heretofore, biomechanical models predicted spinal compression force from posture and hand load without consideration of disc injury status. It is Bayes' theorem that allows for *disc injury*, which is a graph-theoretic descendant of *L5/S1 compression force* on the causal directed path, to influence the estimate of *L5/S1 compression force*. This innovation may be useful in areas such as litigation, for example, where the analysis is being done to estimate whether compression force exceeds a recommended threshold for a specific person who has experienced a prolapsed intervertebral disc.

## Figures and Tables

**Table 1:** Model input posture parameters.

Joint	Angle from horizontal (degrees)	Standard deviation (degrees)
Ankle	82	9.4
Knee	114	9.4
Torso	40	6.3
Shoulder	192	7.9
Elbow	−56	11.8

**Table 2:** Predicted joint moments and L5/S1 disc forces in Newtons.

Quantity	Monte Carlo deterministic	Monte Carlo posture random	Monte Carlo hand load random	Bayesian network deterministic	Bayesian network posture random (SD)	Bayesian network hand load random (SD)
Elbow moment (N.m)	43.5	42.6	43.5	43.4	43.4 (3.9)	43.4 (2.9)
Shoulder moment (N.m)	−39.3	−39.5	−39.3	−39.3	−39.3 (4.0)	−39.3 (2.4)
L5/S1 moment (N.m)	150.9	149.3	150.9	150.9	150.7 (11.8)	150.9 (6.0)
L5/S1 compression (N)	3315.2	3284.1	3316.3	3316.6	3312.6 (212.9)	3316.5 (133.7)
L5/SI shear (N)	682.2	681.4	682.3	682.2	682.2 (12.0)	682.2 (30.0)
